# An automated microprocessor-based spectrophotometric flow-injection analyser

**DOI:** 10.1155/S1463924684000365

**Published:** 1984

**Authors:** Michael Koupparis, Paraskevi Anagnostopoulou

**Affiliations:** Laboratory of Analytical Chemistry University of Athens 104 Solonos Street Athens 106 80 Greece


Journal of Automatic Chemistry, Volume 6, Number 4 (October–December 1984), pages 186–191

### An automated microprocessor-based spectrophotometric flow-injection analyser

**Michael Koupparis and Paraskevi Anagnostopoulou**
*Laboratory of Analytical Chemistry, University of Athens, 104 Solonos Street, Athens 106 80, Greece*

### Introduction

Flow-injection analysis (FIA) has become popular in many laboratories due to its flexibility. FIA is a type of continuous-flow analysis which uses an analytical stream, unsegmented by air bubbles, into which highly reproducible volumes of sample are injected. Application of this principle to automated or semi-automated analysis yields a fast, precise, accurate and extremely versatile system that is simple to operate.

After an initial description by electrochemists [1], the concept of FIA was introduced by two independent groups in 1974. The Danish group (Ruzicka and Hansen [2]), developed the method primarily using instrumentation associated with air-segmented flow analysers (SFA). The American group (Stewart, Beecher and Hare [3]) started with high-performance liquid chromatography (HPLC) components. Thus FIA may be considered a hybrid of HPLC and SFA.

The major benefit of continuous-flow analysis is the capability of performing sample pretreatment functions in-line (dialysis, ion exchange, oxidation, reduction, solvent extraction), in conjunction with a variety of detectors. There are also some inherent advantages in FIA, such as the merging zones, the stopped-flow, the FIA 'titrations' and sample gradients approaches [4–7].

The basic components of an FIA system are a flow-delivery unit, an analytical manifold, a detector and an output-reading device. It is vital that a very reproducible flow is obtained with the delivery unit. Various pump types have been tried, such as syringe, peristaltic, progressive cavity and single or dual piston pumps. The peristaltic pump, which is the most common, is excellent for pumping aqueous fluids but it is not suitable for moving corrosive fluids.

Many kinds of valves have been incorporated in FIA systems to perform sample injection: rotary, solenoid and slider valves for example. The primary considerations in choosing a valve for a particular application are smooth injection and accomodation of the required injection volume.

Critical to the successful operation of an FIA system is the design of the analytical manifold to perform specific functions: sample mixing, reaction and dilution by controlling the sample dispersion for example. The manifold should consist of the sample-treatment components (holding and reaction coils, heating blocks, extraction and dialysis apparatuses, reduction and ion-exchange columns, gradient chamber etc.).

Any detector that can be equipped with a flow-through cell is a candidate for interfacing with FIA. Colorimetry, fluorimetry, flame atomic spectrometry, inductively coupled plasma spectrometry, polarography (differential pulse and differential pulse anodic stripping), ion-selective potentiometry, amperometry and luminescence have been coupled with FIA to form unique analytical systems.

The simplest way of recording the transient signals from the detector is using chart recorders. If the FIA system is used as a workhorse in a busy analytical laboratory, an automatic sampler is desirable. As well as various prototype FIA systems developed by scientists, many commercial FIA analysers are now available: American Research Products Corporation, USA; Breda Scientific, The Netherlands; Fiatron Systems, Inc., USA; Lachat Chemicals, Inc., USA; Mark Instrument Co., USA and Tecator AB, Sweden. All these instruments have advanced facilities but they are expensive.

A relatively low-cost automated spectrophotometric flow-injection analyser, constructed from commercially available components and instruments, is described in this paper. The entire system is automated using a versatile microcomputer.

### Instrumentation

A block diagram of the system configuration is shown in figure 1. It consists of a reagent-delivery unit, a sample-injection system, an analytical manifold, a spectrophotometric detector, an output-reading device and a microcomputer. In operation, the standard or sample solutions are drawn continuously into the sample loop of the rotary valve from the turntable by the sample pump. At preselected time intervals they are injected into the carrier stream. The sample plug is then merged precisely in the analytical manifold with various reagents added in series, and then the reacting mixture flows through the detector. The output of the spectrophotometer is recorded as absorbance peaks (increasing or decreasing) on a chart recorder and is also fed into the microcomputer interface for data reduction and manipulation. The microcomputer controls also the sample-injection system and the turntable.

### *Reagent-delivery unit*

This consists of a four-channel continuously variable speed pump (Sage, Model 375A), with a feedback system to maintain motor speeds. Silicone rubber tubings of 0·5–4·0 mm i.d., used with variable speeds, provide flow rates of 0·8–1020 ml/h. Reagent solutions must be filtered before use to avoid clogging the flow system. The silicone rubber tubings have a typical lifetime of 500 h when pumping aqueous solutions.

### *Sample-injection system*

The sample-injection system comprises a sample pump, a sample-injection valve and an optional sampler. The sample pump used is similar to the reagent-delivery unit and it provides an optimum sample flow of 0·4 ml/min (1·5 mm i.d. tubing with

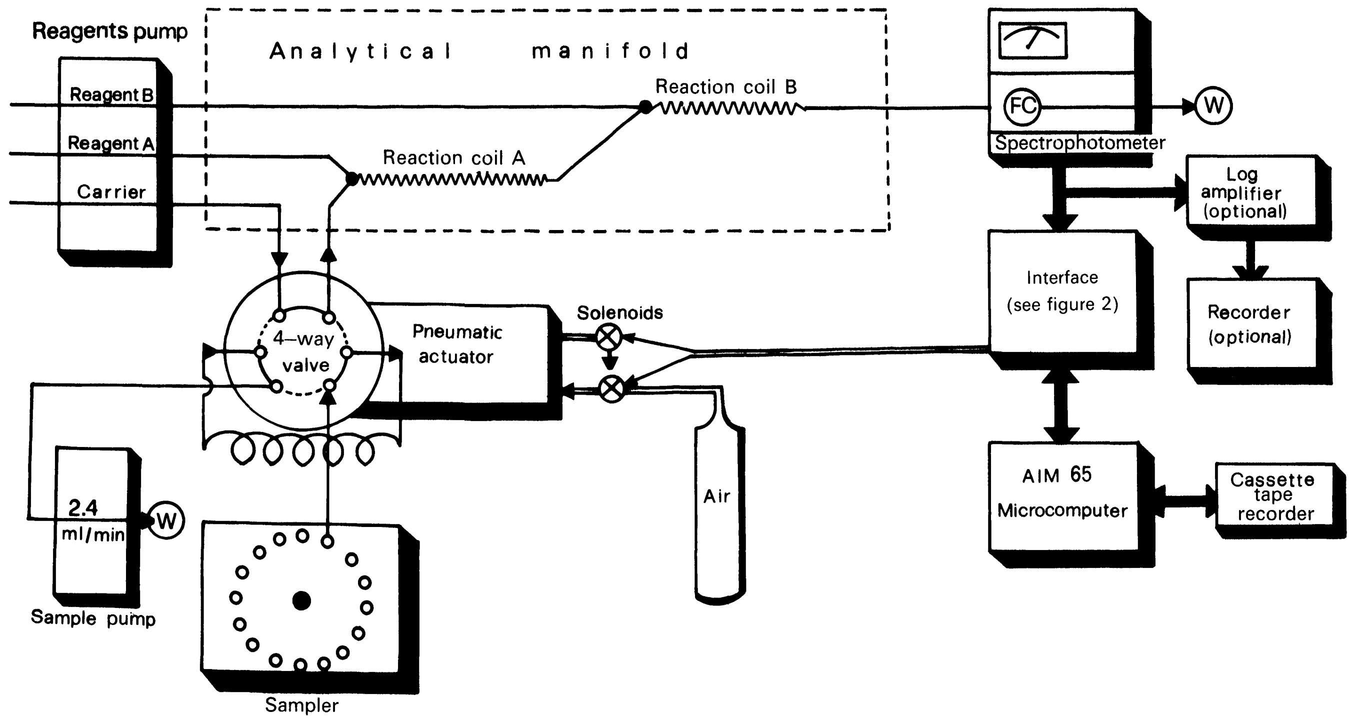

*Figure 1. Block diagram of the University of Athens's flow-injection analyser.*

80% of its maximum speed). A wide range of sample flows for special applications can be selected according to the sample loop size and the load time required. The sample injection valve is a four-way Teflon rotary valve (Rheodyne Type 50) operated with a pneumatic two-position actuator (Rheodyne Model 5001). An air-supply pressure of 30 to 125 psi is required for operation, controlled by the microcomputer through two, three-way solenoids (Angar, 12 V DC, Type 339-V). Sample loops of 0·1–1 ml are used to control the sample volume. An optional sampler (Hook and Tucker, UK, Model A40), modified for automatic control, is used for fully automated operation of the analyser. Manual processing of the solutions to be measured is required otherwise. When turbid solutions are to be measured a small disposable filter (filter for pipette tips, Centaur Chemical Company, USA) is added at the end of the sample probe to avoid any clogging of the flow system.

### *Analytical manifold*

The required analytical manifold for each application is constructed using coils from Teflon tubing, 0·8 mm i.d., around plexiglass rods of 15 mm diameter. Tube end fittings (Lab. Data Control, TEF-107, $\frac{1}{4}$ in, 28 tpi) and Teflon T-connectors (Altex) are used for interconnections. If a high, controlled, temperature is required, the reaction coils and the reservoirs of the reagents are immersed in a standard laboratory water bath.

### *Spectrophotometer detector and recording system*

The spectrophotometer used is the inexpensive Bauch and Lomb, Spectronic 21 model, with solid-state silicon detector, containing a 18 $\mu$l dead volume flow-cell (Hellma Cells Inc., Model 172, 12) with a 10 mm light path. The spectrophotometer output signal (1.000 V for 100% transmittance) is fed into microcomputer's interface and to an optional chart recorder (Health-Schlumberger system) through a logarithmic converter (Pacific Measurements, Inc., Model 1002). The recording system is optional and is helpful when a new automated FIA procedure is being developed. In routine analysis with well-tested procedures, the microcomputer system alone is adequate as the sole reading and data manipulation device.

### *Microcomputer system*

The microcomputer used is the Rockwell AIM 65 (Rockwell International, Anaheim, California, USA) with a visual display and a thermal printer. Input/output operations are performed through a peripheral chip: the R6522 Versatile Interface Adapter (VIA). This chip contains two eight-bit, parallel, input/output ports (I/O) (port A [PA] and port B [PB]). Each bit can be

*Table 1. Memory assignments and functions of the versatile interface adapter (VIA).*

| Location | | Contents | | | |
|---|---|---|---|---|---|
| Hex. | Dec. | Hex. | Dec. | Register | Programmed function |
| A000 | 40960 | | | Port B output Data register (ORB) | |
| A002 | 40962 | 3F | 63 | Port B data direction register (DDRB) | Sets all bits of PB as output except PB6 and PB7 |
| A004 | 40964 | | | T1C-L | Timer 1 counter low byte |
| A005 | 40965 | | | T1C-H | Timer 1 counter high byte |
| A008 | 40968 | | | T2C-L | Timer 2 counter low byte |
| A009 | 40969 | | | T2C-H | Timer 2 counter high byte |
| A00B | 40971 | EO | 224 | Auxiliary control register (ACR) | Sets timer 1 to free-running mode (generates continuous interrupts and a square wave output on PB7). |
| A00D | 40973 | | | Interrupt flag register (IFR) | IFR6 is timer 1 Interrupt flag. It is set by time out of timer 1 and cleared by reading T1C-L or writing T1C-H |

individually selected to be either an input or an output. For this application, only port B is used to output control signals and to input pulses from the voltage-to-frequency converter. The peripheral chip also contains two user-programmable 16-bit timers, which generate, or count, pulses. The peripheral chip's timer 1 functions as a time base and timer 2 as a counter for the V-F converter output.

The manner in which the I/O ports and timers operate is determined by the content of three registers, the data direction registers of ports A and B and the auxiliary control register. Table 1 is a listing of the locations of these registers, the contents which are loaded into the registers in this application, and their function [8]. The registers are loaded with their assigned values using the POKE Basic command at the beginning of the program.

### *System interface*

A diagram of the system interface is shown as figure 2. This consists of a measurement interface to monitor the photometer output, and a control interface for the sample injection valve and the optional sampler. It was built using the Analog-Digital Designer (E and L Instruments, Model ADD 8000) provided with various useful and versatile circuit cards, power supplies and a voltage reference source (VRS).

The measurement interface consists of four operational amplifiers. The OA1 acts as a voltage follower to maintain stable photometer output. In methods with increasing absorbance peaks the photometer output (+10 V for 100% transmittance) is directed to the peak detector input through OA 4 acting as a summing inverter amplifier. This results in a signal at the output which varies from 0 to +10 V. The potential on VRS is adjusted so that, with the photometer calibrated to 100% T, the OA 4 output is zero. In methods with decreasing absorbance peaks the photometer output is directed through OA2 acting as X10 inverter amplifier and OA 3 acting as an inverter, so that the output is compatible with the peak detector. In this case, the input of the peak detector varies from the base-line to +10 V.

The peak detector is the peak detector/sample and hold model 755 made by Hybrid Systems Corporation with an I/O voltage of 0 to +10 V (linearity ±2% FS, tracking rate 0·25 V/μS). The peak detector is reset either by the microcomputer producing a low signal at PB2, or manually with switch S3. The output of the peak detector is inverted by OA5 and is input to a National Semiconductor LM331 voltage-to-frequency converter in its precision configuration [9] (100 KHz full scale, ±0·03% non linearity, ±50 ppm/°C maximum). The pulsed output of the converter is fed to pin 6 of port B through a NAND gate and is counted for a fixed time by the microcomputer's 16-bit timer 2. The fixed time period of measurement is controlled by the square wave output of timer 1 at PB7. By loading FF in T1C-L and T1C-H, a period of 65535 μS is obtained and if this is repeated 10 times an overall measurement period of 655·35 ms can be achieved.

The control interface consists of two identical circuits for the load and inject solenoids of the sample-injection unit and a third, simple circuit for the turntable change. The three control signals, *load inject* and *change*, are connected using buffers to PBO, PB1 and PB3 outputs of port B and are activated by a logic 1 to 0 transition (the 'power on' state of the parallel I/O ports) from the microcomputer. Optoisolators are used to prevent accidental activations by noise. Switch $S_4$, and a similar one for inject solenoid, are used for manual operation.

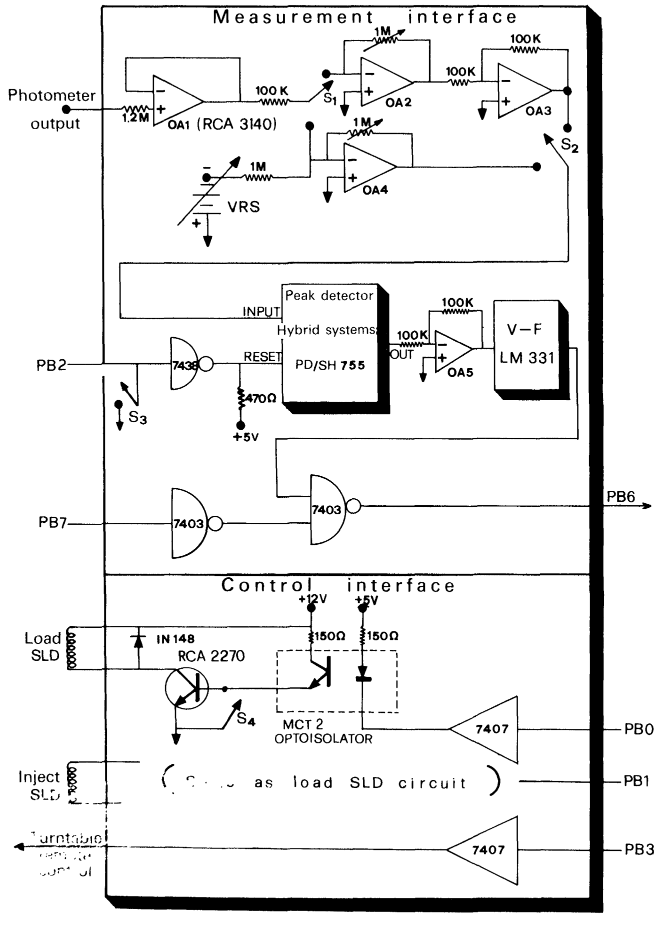

*Figure 2. Diagram of the system interface.*

### System operation

To operate the automated flow-injection analyser, the program is first loaded from the cassette-recorder into the computer's memory. A flow-chart for a typical sequence in a determination is shown in figure 3.

Initially, Basic assigns values to several of the more commonly used variables in the program and sets up the I/O ports and the two timers. Then the operator is prompted by the display to provide information to the program (see figure 3) which includes the type of absorbance peaks (increasing or decreasing). In the increasing peak mode, when the reagents are pumped through the analytical manifold, the photometer is calibrated for 100% T and the VRS of the interface is adjusted so that the output of AO4 (figure 2) is zero. In the decreasing peak mode, the base-line is adjusted at a high level.

The program then sequences through each standard, measuring the base-line, injecting a number of volumes, measuring each absorbance peak, printing the average value and the relative standard deviation and moving the turntable to the next position. The subroutine for the measurement of the absorbance peak is shown as figure 4. In the increasing peak mode, the content of timer 2 counter during the base-line measurement represents the $I_o$ value whereas during a peak measurement it is $I$. So the ΔA peak is equal to $-\log(I/I_o)$. In the decreasing peak mode, $I_o$ is set to be equal to 65535 ($FF), $I_b$ is the counter value for the base-line and $I_p$ for the peak. So the ΔA peak is equal to $-\log((I_p - I_b)/I_o)$.

After the standards have been measured, the microcomputer will calculate the linear least-squares regression line and print its slope, intercept and correlation coefficient. Samples are then

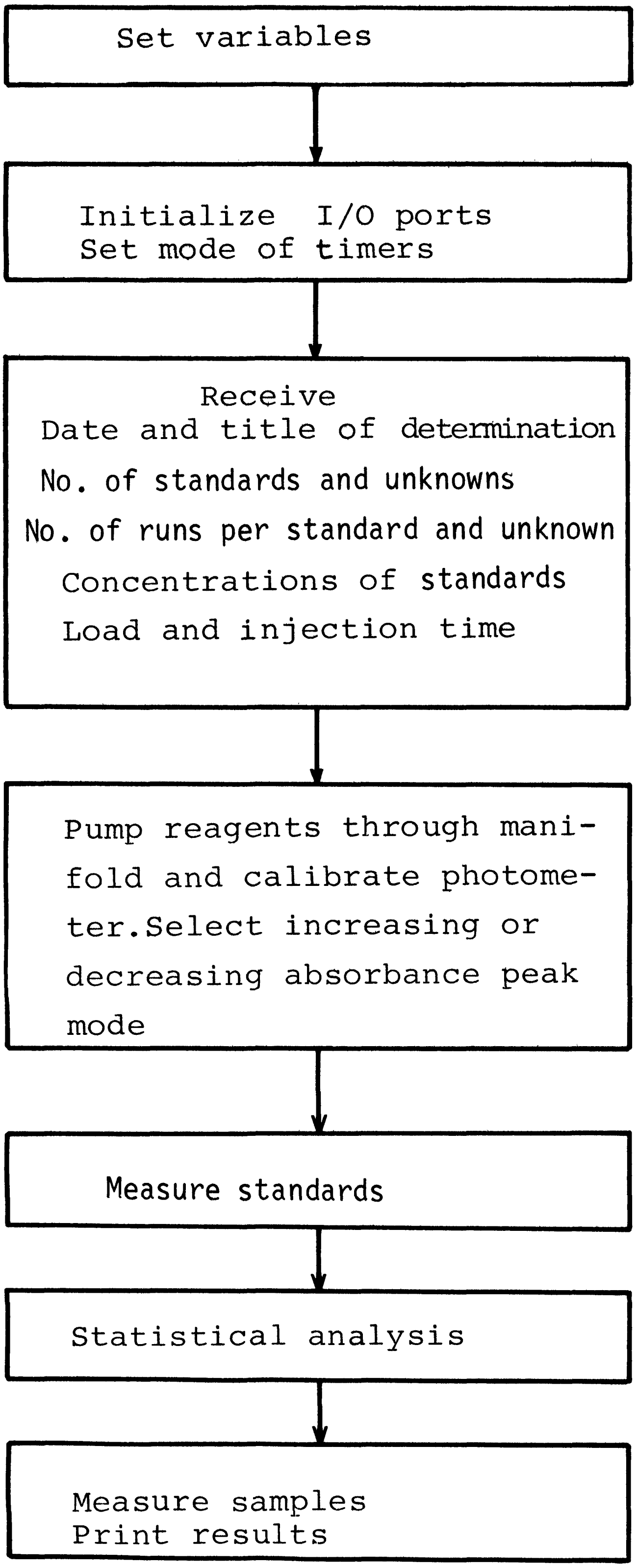

*Figure 3. Flowchart for a typical sequence in a routine determination.*

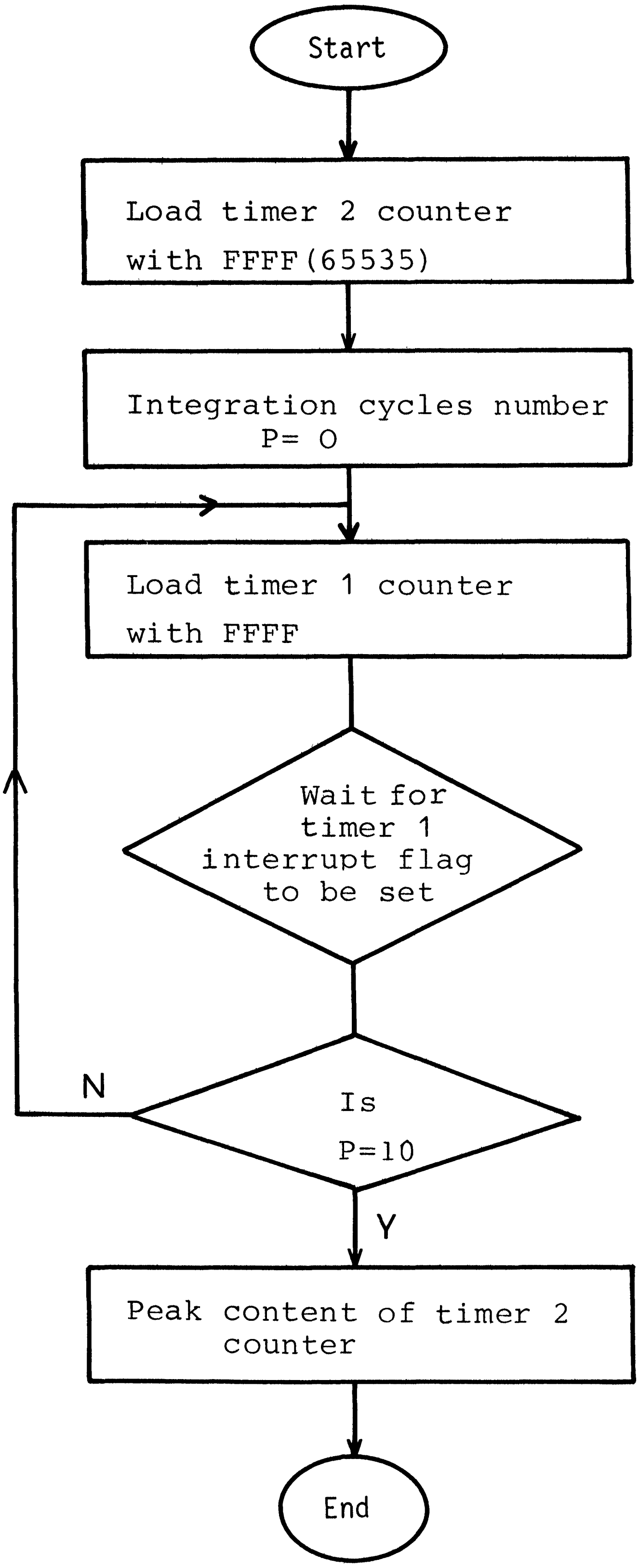

*Figure 4. Flowchart for the absorbance peak measurement subroutine.*

measured, after which the concentration of the analyte in the sample is calculated and printed.

As the base-line is measured before each series of runs of standard or sample, the long-term drift of the system is self-correcting. Control standards can also be included on the turntable after each decade of samples—this increases the accuracy of results.

### Evaluation of the system

Several tests were performed to evaluate the suitability of the system for use in various types of analytical methods. The precision with which solution could be aliquoted, delivered, mixed and measured was determined by three types of test. The first involved the measurement of the precision obtained

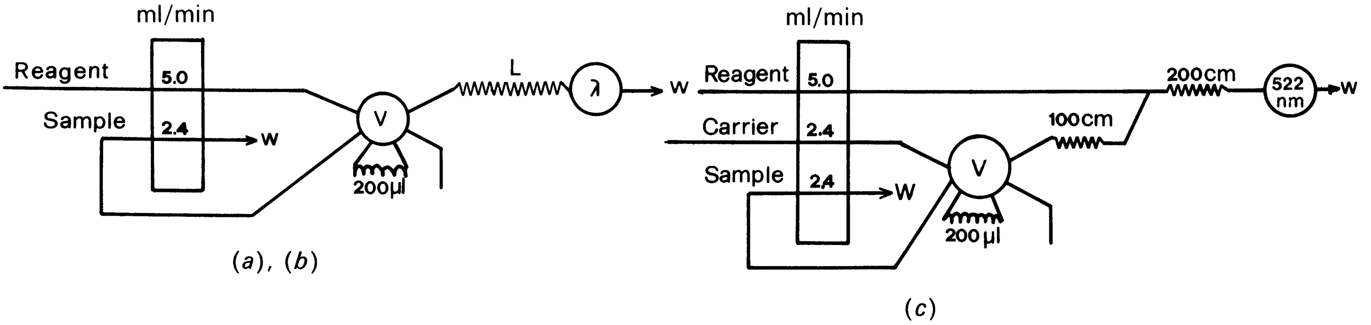

*Figure 5. Analytical manifolds of the FIA system used for: (a) DCPI dilution; reagent: $NaHCO_3$ 210 mg/l, L = 50 cm, λ = 522 nm. (b) Fe (III) determination; reagent: $NH_4SCN$ 0·010 M, samples in 1·0 M $HNO_3$, L = 100 cm, λ = 460 nm. (c) Ascorbic axid determination; reagent: DCPI 1·8 × $10^{-4}$ M in $NaHCO_3$ 210 mg/l, carrier: Oxalic acid 0·05 M, samples in oxalic acid 0·05 M. V-rotating valve, W = waste.*

*Table 2. Reproducibility results for dilution of 2,6-dichlorophenol-indophenol (DCPI) 1·00 × $10^{-4}$ M in α $NaHCO_3$ carrier stream.*

| Series of 10 injections | Average absorbance peak | %RSD |
|---|---|---|
| 1 | 0·3966 | 0·61 |
| 2 | 0·3947 | 0·48 |
| 3 | 0·3940 | 0·69 |
| 4 | 0·3905 | 0·68 |
| 5 | 0·4012 | 0·78 |
| 6 | 0·4008 | 0·56 |
| 7 | 0·4039 | 0·57 |
| 8 | 0·4072 | 0·65 |
| 9 | 0·4060 | 0·60 |
| 10 | 0·3995 | 0·56 |
| Total average | 0·3994 | Total %RSD 1·37 |

Injection time 6 s, loading time 12 s, throughput 200/h.

for dilution of a dye. For this study a solution of 2,6-dichlorophenol-indophenol (DCPI) in a 210 mg/l sodium hydrogen carbonate solution was used. The analytical manifold used is shown in figure 5(*a*). Absorbance measurements were carried out at 520 nm, which is the isosbestic point of DCPI. The relative standard deviations of 10 series of 10 injections each, of a DCPI solution of 1·00 × $10^{-4}$ M, varied from 0·48% to 0·78%. The long-term stability of the system was excellent because of the continuous autocalibration of the base-line. For a time period of 30 min (100 measurements) the total %RSD was 1·37. Similar results obtained with 5·0 × $10^{-5}$ M and 2·0 × $10^{-4}$ M DCPI solutions with %RSD 0·98 and 0·44 respectively (see table 2).

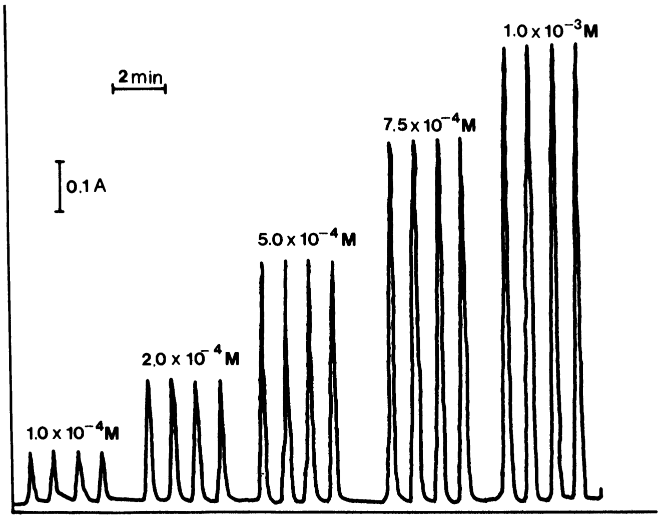

*Figure 6. A recording of decreasing absorbance peaks obtained for the ascorbic acid determination with DCPI.*

*Table 3. Reproducibility results from a Fe(III) determination with $NH_4SCN$ (increasing absorbance peaks).*

| Series of 10 injections | Average absorbance | %RSD |
|---|---|---|
| 1 | 0·5145 | 0·35 |
| 2 | 0·5168 | 0·26 |
| 3 | 0·5187 | 0·27 |
| 4 | 0·5150 | 0·40 |
| 5 | 0·5136 | 0·52 |
| 6 | 0·5133 | 0·34 |
| 7 | 0·5134 | 0·43 |
| 8 | 0·5150 | 0·47 |
| 9 | 0·5156 | 0·57 |
| 10 | 0·5172 | 0·25 |
| Total average | 0·5153 | Total %RSD 0·35 |

Fe(111) = 3·25 × $10^{-4}$ M. Other conditions as in table 2.

*Table 4. Results for an ascorbic acid determination with 2,6-dichlorophenol-indophenol (decreasing absorbance peaks).*

| Ascorbic acid, M | Average peak | %RSD (N = 4) |
|---|---|---|
| 1·00 × $10^{-4}$ | 0·0829 | 1·8 |
| 2·00 × $10^{-4}$ | 0·1604 | 0·74 |
| 5·00 × $10^{-4}$ | 0·3842 | 0·50 |
| 7·50 × $10^{-4}$ | 0·5884 | 0·46 |
| 1·00 × $10^{-3}$ | 0·7621 | 0·37 |

Calibration curve: slope = 759·9, intercept 0·0082, r = 0·9997. Injection time = 15 s, loading time = 30 s, throughput 80/h.

The second test involved an iron (III) determination by the thiocyanate complex formation as an example of increasing absorbance peak FIA procedure. The manifold used is shown in figure 5(*b*) and the results obtained are shown in table 3. For 100 injections (10 series of 10 injections, 30 min operation) of a 3·25 × $10^{-4}$ M Fe(III), the total %RSD was 0·35—showing an excellent long-term stability. Concentrations of 1·95 × $10^{-4}$ M and 5·20 × $10^{-4}$ M gave 0·95 and 0·31 %RSD respectively.

The third test was an ascorbic acid determination using its rapid reaction with 2,6-dichlorophenolindophenol monitored at 520 nm, as an example of a decreasing absorbance peak method. The manifold used is shown in figure 5(*c*), the recorded peaks obtained for a typical calibration curve are shown in figure 6 and the results in table 4. Eighty measurements can be performed per hour.

### Conclusion

From the above examples, it is clear that this automated flow-injection analyser can be used for spectrophotometric methods with accurate and precise results and with a high throughput. The continuous autocalibration of the base-line ensures a high long-term stability. Although a spectrophotometric detector has been described, any other detector could be connected with the FIA analyser given some minor modifications to the interface circuits and the programs.

### References

1. Nagy, G., Feher, Z., and Pungor, E., *Analytica Chimica Acta*, **52** (1970), 47.
2. Ruzicka, J. and Hansen, E. H., *Analytica Chimica Acta*, **78** (1975), 145.
3. Stewart, K. K., Beecher, G. R. and Hare, P. E., *Analytical Biochemistry*, **70** (1976), 167.
4. Ruzicka, J. and Hansen, E. H., *Flow Injection Analysis* (Wiley-Interscience, New York, 1981).
5. Betteridge, D., *Analytical Chemistry*, **19** (1978), 832A.
6. Ranger, C., *Analytical Chemistry*, **53** (1981), 20A.
7. Rocks, B. and Riley, C., *Clinical Chemistry*, **28** (1982), 409.
8. Rockwell International Corporation, *AIM 65 User's Guide* (1978).
9. *Linear Data Book* (National Semiconductor Corporation, 1978), p. 8.

### SAC 86 and 3rd BIENNIAL NATIONAL ATOMIC SPECTROSCOPY SYMPOSIUM—AN INTERNATIONAL CONFERENCE ON ANALYTICAL CHEMISTRY

*20–26 July 1986, University of Bristol, UK*

The Analytical Division of the Royal Society of Chemistry has chosen Bristol as the site for the SAC 86 International Conference on Analytical Chemistry. On this occasion the SAC Conference is to be combined with the Biennial National Atomic Spectroscopy Group of the Royal Society of Chemistry's Analytical Division and the Spectroscopy Group of the Institute of Physics). SAC international conferences are held triennially and still bear the initials of the then Society for Analytical Chemistry which was responsible for their initiation.

The scientific programme will be organized around plenary, invited and contributed papers and posters covering the whole field of analytical chemistry, and all aspects of atomic spectroscopy. As at previous conferences, special symposia on particular analytical themes will be arranged by RSC groups and other associated bodies. The programme will include workshops, where research workers can demonstrate new apparatus and techniques. Update courses are also planned, to provide all-day tutorial and practical demonstration sessions. The latter will be held on the Wednesday, as an alternative to a scientific or cultural visit, or to the 3rd BNASS Programme.

*Techniques—*

Atomic spectrometry
Biochemical methods
Catalytic and kinetic methods
Chromatography
Electroanalytical methods
Electrophoresis
Enzyme techniques
Flow-injection methods
Immunoassay
Mass spectrometry
Microanalysis
Molecular spectroscopy
Photoacoustic spectrometry
Probe methods
Radiochemistry
Sample preparation
Preconcentration and separation
Thermal methods
X-ray methods.

*Materials—*

Agricultural
Atmospheric
Biological and microbiological
Clinical
Environmental
Food and drink
Geological
Industrial
Metallurgical
Particulate
Pharmaceutical
Plastics, rubbers and textiles
Surfaces
Water and effluents.

*Other aspects—*

*Automation and robotics*
Biotechnology
Chemometrics
Consumer protection
Data processing
Education
Harmonization
Historical
Microcomputers and microprocessors
Optical fibres
Quality control
Sensors
The integrated laboratory.

An exhibition of commercial apparatus, equipment and books will be a feature of the Conference. The exhibits will be near the lecture theatre and poster area, and lecture-free times will be organized to allow attendance at the Exhibition.

As for SAC 83 a special issue of *The Analyst*, based on SAC 86/3rd BNASS, will be published, and intending authors who would like their papers to be published therein are invited to include novel or relevant review to satisfy the normal criteria for *The Analyst.*

*The Second Circular will be ready in June 1985; to apply for it and for other information contact The Secretary, Analytical Division, Royal Society of Chemistry, Burlington House, London W1V 0BN.*

